# Evidence for Deficits in the Temporal Attention Span of Poor Readers

**DOI:** 10.1371/journal.pone.0091278

**Published:** 2014-03-20

**Authors:** Troy A. W. Visser

**Affiliations:** School of Psychology, University of Western Australia, Perth, Western Australia, Australia; Centre de Neuroscience Cognitive, France

## Abstract

**Background:**

While poor reading is often associated with phonological deficits, many studies suggest that visual processing might also be impaired. In particular, recent research has indicated that poor readers show impaired spatial visual attention spans in partial and whole report tasks. Given the similarities between competition-based accounts for reduced visual attention span and similar explanations for impairments in sequential object processing, the present work examined whether poor readers show deficits in their “temporal attention span” – that is, their ability to rapidly and accurately process sequences of consecutive target items.

**Methodology/Principal Findings:**

Poor and normal readers monitored a sequential stream of visual items for two (TT condition) or three (TTT condition) consecutive target digits. Target identification was examined using both unconditional and conditional measures of accuracy in order to gauge the overall likelihood of identifying a target and the likelihood of identifying a target given successful identification of previous items. Compared to normal readers, poor readers showed small but consistent deficits in identification across targets whether unconditional or conditional accuracy was used. Additionally, in the TTT condition, final-target conditional accuracy was poorer than unconditional accuracy, particularly for poor readers, suggesting a substantial cost arising from processing the previous two targets that was not present in normal readers.

**Conclusions/Significance:**

Mirroring the differences found between poor and normal readers in spatial visual attention span, the present findings suggest two principal differences between the temporal attention spans of poor and normal readers. First, the consistent pattern of reduced performance across targets suggests increased competition amongst items within the same span for poor readers. Second, the steeper decline in final target performance amongst poor readers in the TTT condition suggests a reduction in the extent of their temporal attention span.

## Introduction

Estimates indicate that between 5–15% of the population suffer from reading disabilities such as dyslexia [1]–[3]. This is a particularly alarming statistic given the increasing dominance of text-based communication such as electronic mail, instant messaging, and online social media in everyday living. The consensus opinion from decades of research is that reading problems are often underpinned by a phonological deficit [4]–[7]. That is, poor readers are less able to parse, process and manipulate phonemic information, leading to reduced phonemic awareness, poorer grapheme-to-phoneme conversion, and ultimately poorer text comprehension.

As well as a phonological deficit, many theorists have proposed that visual processing might be impaired in poor readers. For example, it has been argued that poor readers have a deficiency in their magnocellular system [8], a longer attentional dwell time [9], [10], less-efficient attentional orienting [11]–[12], poorer visual search [13], and less effective inhibitory mechanisms [14], [15]. Notably, however, these visual deficits are often accompanied by phonological problems [16]–[19]. This implies that rather than being independent problems, visual and phonological problems may spring from a common source [16], [20], or at least that visual deficits may not account for unique variance in reading ability beyond known phonological problems.

One visual deficit that can be found independently from phonological problems amongst poor readers is spatial visual attention (VA) span [21]–[24]. Bosse et al. [21] compared children with normal reading ability and dyslexia on variations of partial- and whole-report tasks [25]. In the whole report condition, observers were asked to report all of the letters in a five-item string displayed for 200 ms. In the partial-report condition, observers were asked to report a single letter from the five-item string, indicated by a probe which appeared below the letter-to-be-reported after the offset of the string. Children with dyslexia reported fewer complete five-letter strings in the whole-report task, and fewer letters overall in both the whole- and partial-report tasks. More importantly, while some of the children with dyslexia showed both VA span and phonological deficits, a significant subset showed either phonological deficits alone or VA span deficits alone. This indicated that VA span and phonology make separate contributions to reading impairment, in at least some participants.

To explain the unique contribution of VA span to reading, Bosse et al. [21] suggested that each item in a multi-element visual array competes for access to visual short-term memory [26]. The competitive strength of items is, in turn, determined by the speed with which the item can be processed and the attentional weighting it receives. Given that single-letter reading speed was not significantly different across normal and impaired readers, Bosse et al. [21] concluded that children with dyslexia allocated attention to letter strings less efficiently than normal readers. This conjecture is supported by more recent evidence for reduced information uptake in adults and children with dyslexia [27], [28], and abnormal spatial allocations of attention to letter strings amongst children with dyslexia [23].

The notion that items in multi-element spatial arrays compete for access to VSTM is very similar to some theoretical accounts of item processing during rapid serial visual presentation (RSVP) tasks in which two targets are presented in rapid succession at a single spatial location [29]–[31]. In this so-called attentional blink (AB), identification of the second target (T2) depends on its temporal distance (lag) from the first target (T1), with poorer T2 accuracy at shorter lags and a gradual increase in performance as lag increases. Competition accounts for the AB emphasize competition amongst T1, T2, and the RSVP items directly following them, and suggest that the AB arises because attentional resources are preferentially allocated to T1 and the item directly following it, to the detriment of T2 [29].

The notion that resource competition might underlie item identification in both spatial and temporal arrays and the existence of abnormal VA span in poor readers begs the question of whether poor readers might also allocate attention differently to sequential items presented in a rapid temporal sequence (henceforth, temporal attention span) compared to their normal reading peers. Put differently, one may ask whether poor readers have a more general problem in allocating attention to sequential stimuli in both the spatial and temporal domains. To investigate this issue, a number of studies have examined the AB in impaired readers. In general, results have shown a larger AB in readers with dyslexia [32]–[36] and less-skilled readers [38] (but see [37]). However, as noted by McLean and colleagues, T2 impairments in these studies have often been equivalent across inter-target intervals [39] (but see [33], [34], [38]). This implies that T2 deficits amongst poor readers were not the result of resource competition between T1 and T2, but rather due to a general difficulty processing T2 that occurred independently from T1 processing. On the face of it, then, it would seem that the evidence is inconclusive about whether poor readers show parallel spatial and temporal attention span difficulties.

Before making this conclusion, however, it is important to note that there are significant problems with interpreting results from AB studies as a measure of temporal attention span. Whereas spatial span tasks call for observers to process all items on the display, the AB involves selection of two target items, separated from each other by a number of non-target distractors. Thus, the AB requires not just encoding of potential targets as in spatial span tasks, but also selection of relevant information and inhibition of irrelevant inputs. This point is particularly critical when interpreting existing AB experiments because many researchers have shown that the nature of distractors significantly modulates T2 performance [40]–[44]. The upshot of this is that AB performance likely reflects selection performance just as much, if not more, than inter-item competition, and thus may make a relatively insensitive index of temporal attention span.

In light of this argument, and the lack of clear evidence from existing AB studies on the nature of temporal attention span in poor readers, the present work focuses instead on a modified AB task where targets appeared in direct succession, rather than on measuring T2 performance across a breadth of lags, separated by distractors, as has been done in previous work. The resulting paradigm thus more closely resembled the demands of partial and whole report tasks used to assess spatial VA span. Of course, many AB studies have included some trials with directly consecutive targets. This work has shown T2 performance is largely unimpaired when it follows T1 directly [45], [46], and that this so-called “lag-1 sparing” can extend out to as many as five consecutive targets (“extended sparing”) [40], [47]. Importantly, like spatial VA span, theoretical accounts of sparing suggest T1 initiates an attentional episode that can encompass multiple additional target items as long as they continue to match a target template [40], [42], [46], [48]–[49]. Moreover, there is good evidence for inter-item competition amongst items entering this attentional episode. For example, T2 sparing is often accompanied by significant decrements in T1 accuracy (e.g., [49]–[53]), while experimental manipulations designed to enhance T1 processing often diminish later sparing [54]–[55].

The methodology here closely followed that of Di Lollo et al. [40]. Observers were presented with a series of trials in which two (TT condition) or three (TTT condition) targets appeared in succession. These conditions were designed to elicit lag-1 sparing and extended sparing respectively. In addition, trials with two consecutive targets separated by a single distractor (TDT condition) were also included in order to verify that the presentation conditions used here produced similar results to previous AB studies. The key question was whether the performance of poor readers in the TT and TTT conditions would differ from that of normal readers, implying differences in their temporal span of attention analogous to those found for VA span [21].

## Methods

### Participants

Eighty-five university students (61.2% women; 38.8% men) participated in the study. Ages ranged from 16 to 63 years (*M = *23.24, *Mdn = *21.00, *SD = *9.20; women: *M = *22.92, *Mdn = *21.00, *SD = *9.32; men: *M = *23.73, *Mdn = *21.00, *SD = *9.13). Participants received course credit in exchange for participation in a one hour session. All participants reported normal or corrected-to-normal (i.e., eye glasses or contact lenses) vision and English as their first language.

### Ethics Statement

All experimental procedures were conducted in accordance with the principles expressed in the Declaration of Helsinki, and were approved by the University of Queensland Ethics Committee. Written informed consent was obtained from each participant (and their parents/guardian where applicable) prior to participation.

### Apparatus and Stimuli

#### Reading efficiency

The phonemic decoding efficiency subtest from the Test of Word Reading Efficiency (TOWRE) was used [56]. This test measured observers’ ability to rapidly name as many non-words as possible (top score of 63) without errors in 45 seconds. Non-words were divided into three equal lists printed vertically on a white form. Participants were instructed to read down each list, pronouncing items based on their common sounds, and skipping any items they could not pronounce. Participants were asked to stop reading after 45 seconds and a line was drawn after the last non-word read.

#### Nonverbal intelligence

Nonverbal intelligence was assessed using the nonverbal subtest of the Kaufman Brief Intelligence Test [57]. This subtest was composed of 46 matrices involving meaningful and abstract visual stimuli. Participants were required use fluid thinking and problem solving abilities to identify relationships and complete visual patterns in either 2×2 or 3×3 matrices. Each matrix had one missing item and participants were instructed to choose one of six possible items to complete it. A score out of 46 was recorded.

#### Rapid Automatised Naming (RAN)

Processing speed was assessed using the rapid letter naming subtest from the Comprehensive Test of Phonological Processing [58]. Participants were asked to read aloud, as quickly as possible, 72 items composed of six black randomly arranged letters (*a*, *c*, *k*, *n*, *s*, and *t*) divided into 4 rows and 9 columns on two white pages. Participants were instructed to read across the top row of the first page from left to right before going onto the second row and so on. Once each letter on the first page was named, the same procedure was completed with the second page. The cumulative time taken to name both pages was recorded, along with the number of errors.

#### Temporal attention span task

All stimuli were presented on an Acer AC915 monitor running at a refresh rate of 100 Hz, and connected to a Windows-based computer, using Presentation software (Version 12.4, Neurobehavioral Systems). Targets were the digits 1–9. Distractor items were all letters (except I, O, Q, Z due to their similarity to digits). All items were presented in a light gray (RGB: 170, 170, 170) Arial font (Sz 28; approximately 1°×1° of visual angle) on a black background.

**Figure 1:**
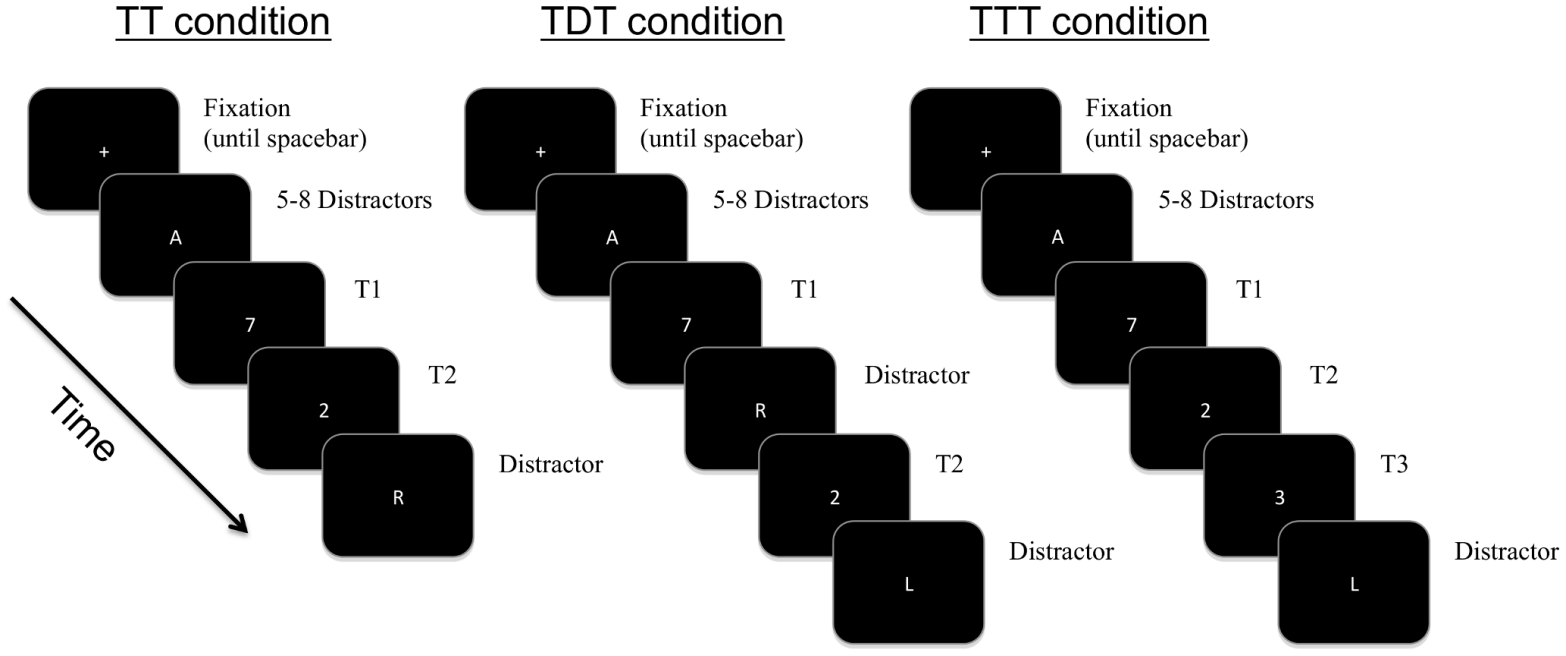
Schematic diagram of TT, TDT, and TTT conditions (not to scale).

A schematic outline of the events on each trial can be seen in Figure 1. Each trial began with a central fixation cross. Participants were then instructed to focus their gaze at fixation, and press the space bar to begin the trial. Following a 300 ms blank screen, a rapid-serial visual presentation (RSVP) stream was presented at fixation. Each item in the stream was presented for 30 ms and followed by a blank screen for 70 ms. After 5 to 8 distractors, T1 was presented, and then followed by: a) T2 (TT condition); b) one distractor and T2 (TDT condition); or, c) T2 and then a third target (T3). Distractors were chosen randomly with replacement, with the proviso that identical distractors could not appear consecutively. The final item in the RSVP was always a distractor that acted as a mask for the last target. After a 200 ms pause, during which the display was blank, participants were prompted to identify the target digits by typing them into the keyboard in any order. Following these responses, the fixation reappeared, indicating the next trial was ready to begin. Each participant completed 25 trials in the TT, TDT, and TTT conditions, yielding a total of 75 trials.

### Procedure

Participants completed the battery of written, verbal and computerized tasks during a one-hour experimental session. All tasks were completed in a quiet dimly-lit room, with participants seated comfortably at a desk or computer workstation. To control for the effects of presentation order, the computerized and non-computerized tasks were counterbalanced separately, with approximately half of participants receiving the non-computerized tasks first, while the other half received the computerized task first.

## Results

Mean target accuracy was calculated without consideration of response order, as is customary in studies of the AB [59] and extended sparing [40]. In addition, both unconditional and conditional accuracy scores were calculated for each target. Unconditional accuracy scores convey the absolute ability of observers to identify each target without consideration of whether they were also able to accurately identify preceding targets. Conditional accuracy, on the other hand, estimates the ability of observers to identify targets, given correct identification of preceding items, and thus provides a way of assessing how processing one target in an attentional episode impacts the ability for additional targets to be processed within that same episode.

To create reading efficiency groups, norms from the TOWRE were used to classify participants as poor or normal readers based on their standard scores. Normal readers were classified as those whose standard scores were greater than 90 (n = 74); poor readers were classified as those whose standard scores fell below the cut-off point (<90) for average reading skill (n = 11). It is worth noting that the proportion of poor readers (12.9%) mirrors the prevalence of reading disabilities in the population. This is in keeping with the fact that the research did not target individuals with reading disabilities, but rather recruited a large sample from the broader undergraduate population in order to capture normal and poor readers.

Mean age, IQ, and RAN scores for both reading groups can be seen in Table 1. Independent-samples t-tests showed no significant differences between age (p>.77) or IQ scores (>.42). However, the difference between mean RAN scores approached significance (p = .07). In addition, there was a significant correlation between TOWRE standard scores and RAN scores (r = −.42, p<.001). For these reasons, we included RAN scores as a covariate in all of the ANOVAs reported below.

**Table 1:** Mean Age, IQ, and RAN scores (in seconds) as a function of reading group (poor vs. normal).

Reading Group	Age	IQ	RAN
Poor	24.00 (2.99)	106.36 (5.23)	27.12 (2.05)
Normal	23.12 (1.06)	110.16 (1.65)	24.33 (0.50)

Numbers in parentheses indicate standard error.

### TDT Condition

**Figure 2:**
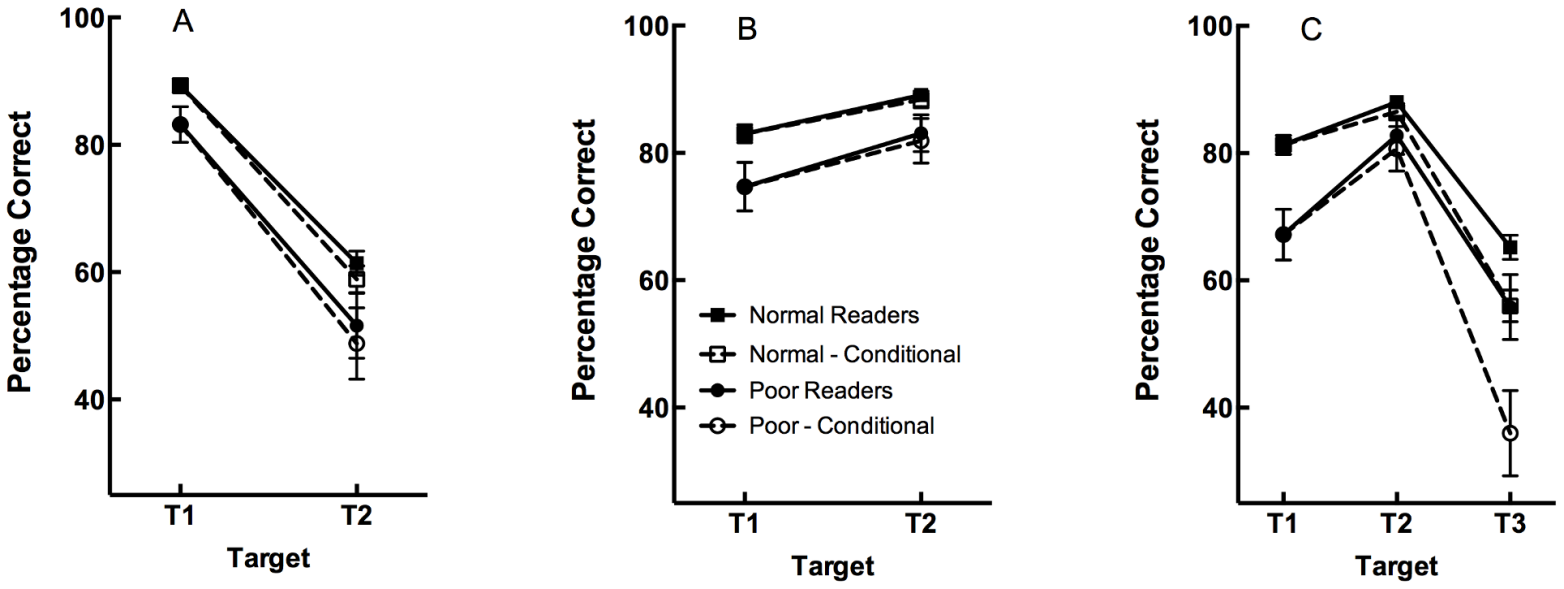
Mean target accuracy in TDT (Panel A), TT (Panel B), and TTT (Panel C) conditions. Dashed lines and open symbols represent conditional target accuracy, while solid symbols and lines represent unconditional target accuracy. Error bars represent one standard error of the mean.

Mean target accuracy scores were analyzed in a 2 (Conditionality: conditional vs. unconditional accuracy) × 2 (Target: T1 vs. T2) × 2 (Reading Group: normal vs. poor) mixed-design ANOVA. Here, and in subsequent analyses, multivariate statistics are reported for within-subjects effects due to unequal sample sizes and violations of sphericity assumptions. The results revealed significant effects of Target, F(1, 82) = 17.12, p<.001, *η_p_* ^2^ = .173, and Reading Group, F(1, 82) = 4.96, p<.03, *η_p_* ^2^ = .057. No other main effects or interactions were significant (all p’s >.19, *η_p_* ^2’^s <.01). Examination of Figure 2 (Panel A) suggests that the main effect of Target reflected generally poorer performance on T2 than T1, consistent with the AB deficit typically obtained when T1 and T2 are separated by one non-target item [40]. These results are broadly in keeping with previous studies that have examined the link between the AB and reading [39], and validate the general paradigm used here. In addition, the main effect of Reading Group indicates that poor readers had lower overall target accuracy. The possible implications of this finding will be discussed in greater detail after reporting the results from the remaining conditions.

### TT Condition

Mean accuracy scores were analyzed in a 2 (Conditionality) × 2 (Target) × 2 (Reading Group) mixed-design ANOVA. This analysis revealed a significant main effect of Reading Group, F(1,82) = 5.14, p<.03, *η_p_* ^2^ = .06, but no other significant main effects or interactions (all p’s >.17, *η_p_* ^2^’s <.03). In sharp contrast to the TDT condition, examination of the results in Figure 2 (Panel B) suggests that sparing was evident across all participants with similar levels of accuracy on T1 and T2. However, the main effect of Target indicates that poor readers again demonstrated significantly impaired target identification across both items.

### TTT Condition

Mean accuracy scores were analyzed in a 2 (Conditionality) × 3 (Target: T1, T2, T3) × 2 (Reading Group) mixed-design ANOVA. This analysis revealed significant main effects of Target, F(2, 81) = 8.25, p<.01, *η_p_* ^2^ = .17, and Reading Group, F(1, 82) = 12.87, p<.001, *η_p_* ^2^ = .14, as well as a Conditionality × Reading Group interaction, F(1, 82) = 8.18, p<.01, *η_p_* ^2^ = .09, and a Conditionality × Target × Reading Group interaction, F(2, 81) = 5.39, p<.01, *η_p_* ^2^ = .12. All other main effects and interactions were non-significant (all p’s >.17, *η_p_* ^2^’s <.03). Examination of Figure 2 (Panel C) suggests that, as in the other conditions, poor readers showed worse performance across all targets, particularly for T1 and T3. This is further evidence for increased competition amongst items in the same attentional episode. Moreover, as suggested by the three-way interaction, poor readers showed greater deficits in T3 performance than normal readers when accuracy scores were calculated conditionally compared to when they were calculated unconditionally (i.e., when T3 accuracy was calculated only on trials where prior targets had been successfully encoded versus trials when accuracy was calculated without regard for whether previous targets had been encoded).

To verify this impression, the three-way interaction was followed with a pair of 3 (Target) × 2 (Reading Group) mixed-design ANOVAs conducted separately for unconditional and conditional accuracy scores. For unconditional scores, this analysis revealed significant main effects of Target, F(2, 81) = 7.69, p<.01, *η_p_* ^2^ = .16, and Reading Group, F(1, 82) = 12.02, p<.01, *η_p_* ^2^ = .13, but no interaction, F(2, 81) = 2.02, p>.14, *η_p_* ^2^ = .05. In contrast, for conditional scores, there were main effects of Target, F(2, 81) = 8.05, p<.01, *η_p_* ^2^ = .17, Reading Group, F(1, 82) = 13.03, p<.01, *η_p_* ^2^ = .14, and a Target ×The follow-up analyses confirm the graphical impression that poor readers showed significantly reduced T3 performance on trials where they accurately encoded T1 and T2 within the same attentional episode, and that this deficit is greater than that found in normal readers. The contrast with results in the TT condition, where no additional deficit was seen for conditional accuracy relative to unconditional accuracy scores, implies that the ability for items to enter the same attentional episode is reduced in poor readers. That is, successful entry of one item into an attentional episode does not impair encoding a further item (TT condition), while the entry of two items into an attentional episode does impair encoding of a further item (TTT condition).

## Discussion

Reading problems are a significant disability that has persisted at relatively high levels for the last thirty years. Moreover, with the dramatic increase in the importance of text-based media in the past decade, this is a problem that is likely to have even greater consequences in the future. While, a large body of research has shown a connection between phonological processing and reading impairment, more recent experiments have also shown significant deficits in spatial VA span amongst impaired readers [21]–[23] that predict reading performance over and above the presence of phonological deficits.

The present work examined whether deficits similar to those seen in spatial VA span could be found in the temporal attention span of poor readers. A number of studies have examined a similar issue in the past using the AB paradigm. However, the results have been distinctly mixed [39], and it is unclear whether the AB taps into a temporal analog of the spatial VA span, because targets are often interspersed with distractors, requiring additional processes of selection and/or inhibition not required in spatial VA tasks, and because processing occurs across significantly longer time spans than in tasks used to estimate VA span [27].

To address these shortcomings, the present work employed a modified AB paradigm in which observers viewed two (TT condition) or three (TTT condition) consecutive targets, as well as control trials in which two targets were separated by a single distractor (TDT condition) as in the conventional AB. Of chief interest was whether poor readers would show reduced target accuracy across consecutive targets, indicating a difference in their temporal span of attention. Consistent with this possibility, two key results emerged. First, across all conditions, poor readers showed an overall reduction in target accuracy compared to their normal-reading peers. Second, when presented with three consecutive targets, poor readers showed distinctly poorer T3 performance when accuracy was calculated conditional on accurate identification of preceding targets compared to when accuracy was calculated without consideration of accuracy on preceding targets (unconditional accuracy). This implies that poor reader’s ability to process successive inputs within the same attentional episode is significantly curtailed beyond two items, indicating a temporal span deficit analogous to the VA span deficit shown in previous studies [21]–[25].

What are the origins of this temporal span deficit? Theoretical accounts suggest that VA span is determined by inter-item competition for entry into VSTM and that impaired readers differentially distribute attention amongst items compared to normal readers [26] as a result of reductions in processing speed and abnormal allocation of attention across items [23], [27], [28]. Theoretical accounts from the AB literature also suggest that inter-item competition plays a key role in determining consecutive target performance [29]. This conjecture has been bolstered by numerous studies showing changes in performance on one target are mirrored by concomitant variations in performance on other targets when items are presented in direct sequence [49]–[55]. Taken together, then, it seems reasonable to hypothesize that reductions in temporal span were the result of increased inter-item competition in poor readers.

One possible source of increased competition would arise if poorer readers were less efficient at filtering out the distractors that preceded target sequences in the TT and TTT conditions. This would result in additional distractor items competing for VSTM access, thereby reducing target accuracy. While this is plausible, several previous studies have failed to show reduced T1 performance in the AB deficit amongst poor readers [10], [38] even when target-distractor similarity was quite high, implying that their target selection was unimpaired. In addition, this account would seem to predict a larger T2 accuracy difference between poor and normal readers in the TDT condition relative to the TT condition, given the additional distractor interposed between T1 and T2. However, comparison of performance across these conditions shows no interaction between condition and reading ability, F(1, 83) = .21, p>.64, *η_p_* ^2^<.01. Lastly, it is difficult to explain how inadvertent processing of distractors presented prior to T1 would yield a significant difference between reading groups on T3 in the TTT condition, but not on T2.

A second possible explanation for increased competition amongst poor readers is that they have slower processing speed, rendering them less able to adequately process the rapid visual inputs presented on each trial. While there is evidence for the role of processing speed in VA span, at least two points would seem to countermand this as a possible explanation here. First, previous work has suggested that processing speed is unrelated to the magnitude of the AB per se, but rather to the ability of observers to filter out distractors [60]. As noted above, given that there is little evidence that poor readers are less able to filter out distractors, either in the present study or in previous experiments, this would imply that differences in processing speed cannot account for the present differences in temporal attention span. More importantly, analyses were conducted using RAN performance as a covariate, thus obviating processing speed as the source of statistical differences between reading groups.

Another option to consider is that competition is less important in determining temporal attention span. Rather, reduced target accuracy reflects impairments in working memory capacity. Such reductions have been repeatedly demonstrated amongst poor readers [61]–[64]. Moreover, a reduced ability to store information would provide a plausible reason for the failure to report T3 in the TTT condition. One way to test this explanation is by comparing the TT and TTT conditions. If inter-target competition was responsible for reduced T3 accuracy amongst poor readers then other targets in the TTT condition should also show performance changes. On the other hand, if the decrease in T3 performance were simply due to the addition of another target, thereby exceeding working memory capacity, then we might expect only T3 accuracy to suffer. Consistent with the former option, T1 performance was significantly lower amongst poor readers in the TTT condition compared to the TT condition, t(10) = 2.44, p<.04. It is also notable that T2 accuracy amongst poor readers was not significantly different in the TTT condition compared to the TT condition, t(10) = 0.45, p>.66. This rules out the option that the addition of a third target led to a global decline in working memory capacity that affected encoding of all targets amongst poor readers.

Having considered explanations for the present results, it is also important to compare these findings to outcomes of previous studies. One similar study is that of Lassus-Sangosse, N’guyen-Morel & Valdois who presented multiple consecutive target items in a single spatial location [65]. Unlike the current findings, however, they obtained no differences between normal readers and those with dyslexia. Critically, Lassus-Sangosse et al. [65] used a much longer inter-target interval of 200 ms between items. This may be particularly important because studies of the temporal parameters of lag-1 sparing suggest that this longer interval is outside the temporal span of a single attentional episode [31].

Additionally, several previous studies that have examined the relationship between AB and reading have contained lag-1 trials with consecutive targets as in the TT condition used here [34], [38]. To my knowledge, only Laasonen et al. [34] compared lag-1 sparing across reading groups, and they found no differences. However, given that there was also no evidence for sparing across any of the reading groups in this study, this result is difficult to interpret in the present context. In general, it would seem that additional work would be important to further examine the processing of consecutive targets across different experimental contexts.

Although the present findings are intriguing, it is also critical to consider potential limitations of the current study. One set of limitations stem from the characteristics of the participants which included eleven poor readers drawn from a pool of undergraduate psychology students who were predominately young adult, relatively affluent, female and had somewhat higher IQs than those in the population. This opens up the possibility that the results here would not extend to larger samples with more diverse characteristics and academic backgrounds. That said, while such generalizability must be demonstrated in future work, there are some suggestions that this is likely to occur. First, despite the small sample size, the differences seen between poor and normal readers were highly statistically significant and obtained reliably across experimental conditions. Second, the sample size of poor readers is consistent with studies using similar paradigms [10], [32], [38], [66], [67] and studies examining spatial VA span [22], [23], [27], [33]. Finally, previous work has shown considerable overlap between RSVP task performance in poor readers drawn from university, community and child samples [10], [38], [39], suggesting broad similarities exist between participants with diverse backgrounds, ages, and cognitive abilities.

Another potential limitation of the study is that it did not assess whether poor readers met criteria for a diagnosis of dyslexia nor whether they had other co-morbid clinical conditions, such as attention-deficit-hyperactivity disorder or depression that might have contributed to task performance. As a result, the present experiment cannot speak to whether temporal attention span would also be impaired in a group of readers with dyslexia compared to their normal-reading peers, nor whether temporal attention span is related directly to reading or indirectly via a comorbid condition. Clearly, these are both important questions to be answered in future research if we are to fully characterize the relationship between temporal attention span and reading disability. However, the unique evidence presented here for a link between reductions in temporal attention span and reading proficiency still has important implications for understanding poor reading. In addition, as noted above, past studies suggest broad similarities in RSVP task performance across poor readers and readers with dyslexia. This bolsters the likelihood that temporal attention span deficits will also be implicated other instances of reading impairment.

In sum, while it is widely accepted that reading impairments often have a phonological basis, there is persistent and growing evidence that visual processes may also play a role in many instances. In particular, recent evidence has suggested that poor readers often have impaired spatial VA spans, even when no phonological impairment is present. This work provides the first evidence that poor readers may have analogous impairments in temporal attention span. In particular, the results here suggest a reduced capacity for simultaneous item processing within attentional episodes arising from increased inter-item competition. It remains for future work to confirm, clarify and expand upon these initial indications, and examine how temporal attention span impairments may impact upon reading performance in more severely impaired readers.
